# Unrealistic expectations or hopeless actions: The importance of a comprehensive survival strategy to improve cardiac arrest outcomes

**DOI:** 10.1016/j.resplu.2023.100408

**Published:** 2023-06-06

**Authors:** Laurent Suppan, Roman Burkart, On behalf of the Swiss Resuscitation Council

**Affiliations:** Swiss Resuscitation Council, Bern, Switzerland; Division of Emergency Medicine, Department of Anesthesiology, Clinical Pharmacology, Intensive Care and Emergency Medicine, University of Geneva, Hospitals and Faculty of Medicine, Geneva, Switzerland; Swiss Resuscitation Council, Bern, Switzerland; Interverband für Rettungswesen IVR-IAS, Switzerland

*To the Editor,*

We read with great interest the article by Gross et al. reporting the results of a national survey regarding do-not-resuscitate preferences.^1^ On behalf of the Swiss Resuscitation Council (SRC), we congratulate the authors for this thought-provoking article. The SRC strongly supports all research and communication interventions aiming at improving informed consent and the drafting of advance directives.^2^ A clear understanding of the risks and benefits of medical procedures is indeed necessary to make sound, informed decision, and this is even more important when considering out-of-hospital cardiac arrest (OHCA).

While we agree that media often give a falsely optimistic representation of OHCA outcomes, the only clinical vignette presented by Gross et al. erred somewhat on the other side by asking the participant to impersonate a 70-year-old person with a 10-minute no-flow time. The participants acknowledged the poor prognosis linked to this delay, with only 59.5% responding that they would rather undergo resuscitation under such circumstances. However, 79.7% of participants responded that they would want to be resuscitated if they presented an OHCA in their current situation. The authors concluded that participant willingness to undergo resuscitation was linked to their overestimation of positive outcomes.^1^

However, the 8.5% survival rate with good cerebral performance category scores (1 or 2), which was used as reference,^3^ can be considered conservative and does not necessarily apply to Switzerland. Indeed, the Swiss Registry of Cardiac Arrest (SWISSRECA) shows that this rate is higher than 32% in specific subsets of OHCA patients.^4^ Achieving such rates requires the immediate provision of basic life support after collapse,^5^ and public health strategies need to be established to allow this. Other patient groups have much lower survival rates with good neurological outcomes. It must however be emphasized that all cardiac arrest outcome rates vary widely from one region to another; in North America, survival rates varied from 0% to 28.9% according to the EMS agency considered.^6^ Therefore, we should strive to find solutions to improve these rates rather than focus on bleak numbers.

Since estimating the probability of survival with good neurological outcome is a difficult and complex task, the burden of deciding whether to continue or withhold resuscitation should rest with professional caregivers, both inside and outside the hospital. In Switzerland, the performance of these providers is regularly assessed through the SWISSRECA. The last report showed that an important triage effort already took place in the prehospital phase, with less than half of all OHCA patients transported to the hospital.^4^ Furthermore, 74.4% of patients discharged from a Swiss hospital after OHCA had a CPC score of 1 (20.4% presented a score of 2). These figures show that intensive care teams are remarkably effective at identifying patients with a good neurological prognosis.

In conclusion, the SRC recommends endorsing comprehensive, evidence-based strategies to improve OHCA outcomes. Such strategies allow the training and recruitment of first aid providers without altering the selection of patients in whom resuscitation maneuvers should be withheld.

## Declaration of Generative AI and AI assisted technologies in the writing process

The authors did not use AI to create this manuscript.

## Funding

Not applicable.

## Declaration of Competing Interest

The authors declare that they have no known competing financial interests or personal relationships that could have appeared to influence the work reported in this paper.
